# Co‐Designing Local Collaboration Between Social Services, Primary Healthcare, and the Third Sector—A Realist Process Evaluation

**DOI:** 10.1111/hex.70672

**Published:** 2026-04-11

**Authors:** Maria Flink, Karin Johansson, Christofer Lindgren, Sarah Wallcook, Charlotte Klinga, Bettina Meinow, Ida Goliath, Helena Strehlenert

**Affiliations:** ^1^ Research and Development Unit for Elderly Persons (FOU nu), Region Stockholm Stockholm Sweden; ^2^ Department of Neurobiology, Care Sciences and Society Karolinska Institutet Stockholm Sweden; ^3^ Nestor Research and Development Centre Haninge Sweden; ^4^ Research and Development Unit for the Social Services and Adjacent Health Care (FoU Nordost) Stockholm Sweden; ^5^ Stockholm Gerontology Research Center Stockholm Sweden; ^6^ Department of Learning, Informatics, Management and Ethics Karolinska Institutet Stockholm Sweden; ^7^ Centre for Person‐Centred Care University of Gothenburg Gothenburg Sweden; ^8^ Academic Primary Healthcare Centre Stockholm Health Care Services Stockholm Sweden; ^9^ Department of Neurobiology, Care Sciences and Society, Aging Research Center, Karolinska Institutet Stockholm & Stockholm University Stockholm Sweden

## Abstract

**Introduction:**

Co‐design is increasingly used to develop research interventions and community services, highlighting the need for a deeper understanding of how this method drives change. This study evaluates the contextual factors and mechanisms shaping a co‐design process intended to foster local collaboration between social services, primary care and the third sector.

**Methods:**

A realist process evaluation was conducted in two stages: (1) formulation of an initial programme theory (IPT) consisting of Intervention‐Context‐Actor‐Mechanism‐Outcome (ICAMO) configurations, and (2) testing and refinement of the IPT. The IPT drew on comprehensive documentation from the planning and implementation phases of the co‐design process. To test and refine the IPT, six group interviews were conducted using realist interviewing and analysed deductively using IPT‐derived categories.

**Results:**

Three interconnected ICAMO configurations were identified. The first concerns stakeholders' assessment of the potential value of participating in the co‐design process and their decision to engage. The second highlights how emerging trust enabled respectful and constructive dialogue. The third focuses on the facilitation of ideation and prototyping of goals and terms for collaboration. Key mechanisms include sensing an opportunity for change, building trust, and experiencing group agency.

**Conclusion:**

Successful co‐design requires facilitation that not only considers contextual factors but is also perceived as impartial, as such neutrality is vital for establishing trust. Constructive participation depends on representatives who combine relevant practical knowledge, a sense of opportunity for change, and the mandate or organisational conditions to act on outcomes. Continuity in participation, ensuring that the same individuals remain involved throughout the process, is also crucial for maintaining competence and for building the relationships and trust needed to develop shared goals and collaborative structures.

**Patient or Public Contribution:**

Patients and the public were not directly involved in the design, conduct or analysis of this study. However, this evaluation examined a co‐design process involving third‐sector organisations, including older adults. A public advisory board contributed to the broader participatory action research project.

## 1 Background

Collaboration among community actors to prevent health decline in older adults has become a governmental priority in Sweden and globally [1, 2], driven by specialisation of services and professionalisation [3], and an aging population in need of multiple services. Collaboration is a type of integration between organisations, relying on agreements, adjustments and a willingness to work together [3]. This dynamic and interactive process requires work across professional and organisational boundaries to achieve common goals, utilising the skills and competencies of all partners [4]. Although collaboration has been a top priority for policymakers for decades, evidence of whether collaboration in welfare systems can improve outcomes is scarce [5, 6]. Limited availability of evidence may partly be due to contextual factors, highlighting the need for further research and a deeper understanding of which types of collaboration are effective and under what circumstances [5]. Although the importance of community engagement in collaborative efforts is well established [5], little insight remains into how collaboration between third sector and other welfare stakeholders is initiated and maintained over time [7]. One way to foster sustainable collaboration in this area is by actively incorporating the perspectives of all involved stakeholders—an approach that can be facilitated through co‐design processes.

Co‐design, along with related concepts such as co‐production and co‐creation, is increasingly recognised as a promising approach for developing innovative ways of working [8]. Co‐design is a process grounded in collective creativity where facilitators and stakeholders work together to achieve shared goals, often resulting in products or services that would be difficult or impossible for any single stakeholder to attain alone [9]. ‘Stakeholders’ within co‐design are generally referred to as service‐users, implying both people with lived experience, such as patients, representatives from patient organisations, professionals or managers [10]. In co‐design, the collective efforts of diverse stakeholders are typically guided by a facilitator, who may be a researcher or a trained designer [9]. The facilitator leads the participants through a structured process that includes various design phases. Commonly referenced co‐design process models include the Double Diamond, Design thinking, and Experience‐Based Co‐design [10, 11, 12, 13]. These models share several core characteristics, including an emphasis on building a shared understanding of the issue at hand, engaging participants in creative development activities, and using prototyping to refine products or services. As interest in co‐design has grown within research, attention has been drawn to the limited scientific depth in studies exploring its facilitation, highlighting the need for more empirical research in this area [14]. At the same time, its increasing use in both practice and research has prompted calls to explore the underlying values, principles and outcomes of co‐design to better understand the contexts in which it is most useful [8, 15]. Although co‐design is generally associated with positive outcomes, the lack of common strategies and standardised models for evaluating its effects remains a challenge [16]. This limitation reflects broader difficulties in assessing complex interventions and has sparked a demand for new evaluation approaches that consider not only outcomes, but also for whom, when, and under what conditions such interventions are effective.

Process evaluations that explore the interplay between intervention, context and mechanisms have become standard practice for assessing complex interventions such as co‐design processes [17, 18]. Building on this, realist process evaluation is recommended for organisational interventions as it offers deeper insights into how underlying mechanisms generate the outcomes within specific contexts [19, 20]. Realist studies argue that complex interventions work differently in different contexts, which means that an intervention can be successful in one setting but less effective in another, depending on multiple contextual factors such as organisational culture, available resources, stakeholder engagement, and external influences that shape both implementation and outcomes [21, 22]. Realist process evaluations aim to examine how complex interventions are implemented in practice, what outcomes they generate, and which contextual factors and underlying mechanisms influence the implementation process. These evaluations are guided by an Initial Programme Theory (IPT)—an explicit hypothesis about how an intervention is expected to work, based on the interaction between context (C), mechanisms (M) and outcomes (O) [22]. Recent research suggests that a more comprehensive understanding of complex interventions can be achieved by expanding Pawson and Tilley's [22] CMO configuration to also include the intervention itself (I) and the actors involved (A), resulting in the ICAMO heuristic tool [23, 24]. The key concepts of an ICAMO configuration are defined as follows: an *Intervention* refers to a combination of programme elements or strategies designed to produce behavioural change or improve health among individuals or groups; *Context* denotes the conditions that enable or constrain the activation of programme mechanisms; an *Actor* is the individual, group, or institution involved in implementing the intervention and shaping its outcomes; a *Mechanism* refers to the underlying determinants of social behaviour that are triggered in particular contexts; and an *Outcome* is both the immediate effect generated by the programme activities, the behaviour changes following immediate effect, and the long‐term outcomes generated from these behaviour changes [23, 24]. The ICAMO tool underscores the dynamic relationships among the key concepts by highlighting that outcomes depend on mechanisms being activated under appropriate contextual conditions and through the participation of the key actors. The ICAMO is an analytical tool that helps to deepen understanding of how co‐design, as a complex intervention, tackles the wicked challenge of collaboration within welfare systems by revealing the underlying mechanisms that drive outcomes in specific contexts.

The purpose of this study is to evaluate contextual factors and mechanisms of importance in a co‐design process aimed at developing collaboration between social services, primary care and the third sector.

## 2 Methods

This study was part of the participatory action research project SAMSAS, which was conducted in Stockholm County, Sweden. The authors designed and facilitated a co‐design process (the ‘intervention’) involving representatives from social services, primary health care, and the third sector to create local goals and terms of collaboration aimed at preventing health deterioration among older adults. A realist process evaluation was conducted to highlight contextual factors and mechanisms underlying the co‐design process. Ethical approval was granted by the Swedish Ethical Review Authority (registration number 2021‐04645).

### 2.1 Setting

Sweden has a decentralised and fragmented health‐ and social care system [25]. It relies on collaboration between various actors, such as municipalities, regions and third sector organisations, each responsible for different components of the system [25]. The 290 municipalities are responsible for social services and are legally obliged to support their citizens and provide opportunities for an independent, active and meaningful community life for older adults. Social services' preventive interventions include, e.g., activities at senior centres and services targeting individuals with specific needs, e.g., digital assistance services or support for informal caregivers. The 21 regions are responsible for specialised care and most of the primary care. Primary care handles several preventive health care interventions, including screening for early detection of physical and mental health issues. Third sector organisations target older adults through both professional and non‐professional non‐profit organisations, such as senior citizens' associations, the Red Cross, or religious communities. The third sector offers a wide array of preventive interventions including meeting venues and platforms, and social activities, often relying on voluntary commitment. Municipal, regional and third sector stakeholders thus share some common goals for prevention but also operate according to different logics, which may impede effective collaboration between them.

### 2.2 Intervention

The co‐design process was conducted in three municipalities (hereafter referred to as cases) in a series of workshops between March 2022 and June 2023. The process included a recruitment phase and six onsite, in‐person workshops per case to map the current situation and develop shared goals and terms of collaboration. Each series of 2‐h workshops were conducted over 5–6 months, i.e., every 3–4 weeks, in each case. The co‐design process for the cases was partly conducted in succession, with a partial overlap for Cases B and C, which made it possible to draw on the experiences from previous workshops to some extent. The municipal Prevention units were actively involved in identifying and recruiting potential local collaborators from social services, primary health care and third sector organisations. These units, operating within older adult social services, have been recently established to address the current challenge of preventing conditions that could impact the health and quality of life of older adults. They provide a wide range of services for older adults living at home and they have knowledge of, and often maintain contact with, primary care and third sector organisations in the local community. Each participating organisation was encouraged to appoint, if possible, at least two representatives to take part in the co‐design process. The group in each case consisted of 12–21 participants, representing a variety of roles and functions (Table 1), such as assistant nurses, social workers, librarian, deacon, health educator and home care team leader. In all cases, the representatives from the senior citizen organisations were older adults with their own experiences of participating in activities to prevent health deterioration.

**Table 1:** Participants in the co‐design groups across the three cases.

	Municipal and social services, e.g., activity centre, home care, family caregiver support, library.	Primary health care, e.g., primary health care centre, home health care.	Third sector organisations, e.g., senior citizen associations, volunteer organisations, church.	Total
Case A	6	1	5	12
Case B	7	2	3	12
Case C	14	0	7	21

All authors contributed to the design of the workshops that formed the co‐design process. Guided by the Stanford Design Thinking model [12], the workshops followed the phases empathise, define, ideate and prototype (Table 2). The *Empathise* phase focused on fostering trust and an inclusive environment where participants could build relationships and voice their perspectives. In the *Define* phase, participants developed a shared understanding of the problem, while the *Ideate* phase was dedicated to generating potential solutions. Finally, during the *Prototype* phase, these ideas were refined into specific and practical solutions that could be implemented in participants' everyday work. The workshops were facilitated through a combination of small group and plenary discussions.

**Table 2:** Overview of the intervention and facilitators' roles.

Co‐design phase	Focus of co‐design	Activities
Planning	*Recruitment of stakeholders*	– Identify and discuss potential stakeholders to invite – Draft invitation to the co‐design process – Invite local collaborators
Empathise and Define	*Workshop 1* – Introduce research project and Prevention unit's purpose in joining the project – Map current prevention activities and collaboration – Discuss potential foci for collaboration	– Icebreaker activities – Brainstorming – Reverse brainstorming – Brainwriting – Interviewing in pairs – Discussions in mixed or stakeholder‐specific groups of varying sizes – Facilitator feedback, including summary of discussions – Open voting – In between workshops: compile and communicate documentation
	*Workshop 2* – Get an overview of the different perspectives and challenges related to prevention and collaboration	
Ideate	*Workshop 3* – Specify and motivate foci for the collaboration – Prioritise options and jointly select the focus for the current co‐design process	
	*Workshop 4* – Discuss and formulate a shared goal – Generate ideas for how to develop collaboration to reach the goal	
Prototype	*Workshop 5* – Continue to develop/revise shared goal for collaboration – Develop draft on goals and terms for collaboration	
	*Workshop 6* – Specify drafts on goals and terms of collaboration – Plan for continued collaboration	

A small team of researchers (authors MF, HS, KJ), all with previous experience of leading co‐design and creative workshops with participants from multiple organisations, facilitated the workshops. Between sessions, these facilitators compiled notes from the previous workshop, shared them with participants via email and prepared for the next session. Preparation included, e.g., selecting creative methods (exemplified in Table 2) to support the co‐design process. In all three cases, participants first discussed current challenges to collaboration, then developed local goals and terms for collaboration, laying the foundation for their own continued collaboration beyond the workshop series.

### 2.3 Study Design

This realist process evaluation [22] was conducted in two stages. First, we formulated an IPT of the intervention, which included Intervention‐Context‐Actors‐Mechanism‐Outcome (ICAMO) configurations. Second, we tested, refined and validated the IPT with relevant stakeholders [23, 24].

### 2.4 Data Collection and Analysis

#### 2.4.1 Stage 1: Formulating the Initial Programme Theory

The IPT was formulated using data comprising comprehensive documentation from the planning and implementation of the co‐design process, participant interviews and relevant academic and grey literature on co‐design and collaboration [8, 14, 15, 16, 26, 27, 28, 29, 30, 31, 32, 33]. The documentation from the planning and implementation of the co‐design process included workshop agendas, slides, worksheets, audio recordings of workshops, and facilitator reflection sessions. After each workshop, the facilitation team held semi‐structured reflection sessions (*n* = 18, six per case, 15–30 min) to discuss co‐design process dynamics, participant responses, and goal attainment. Thereto, 48 short participant interviews (Case A *n* = 18, Case B *n* = 14, Case C *n* = 16) with a total of 58 persons were conducted between workshops, with individuals or small stakeholder groups of two to four participants, lasting 10–20 min via video or phone, to capture their reflections on the process and group interactions. All reflections and interviews were recorded and transcribed.

Authors MF and HS met weekly to synthesise insights from data and developed the IPT through retroductive reasoning [34], a process that moves iteratively between data and theory to identify patterns within and across cases. The documents, the transcribed reflections and interviews, and the literature were read in parallel throughout this process. In a joint document, notes were made on hypotheses about contextual factors, actors, mechanisms and outcomes on how and why the co‐design intervention was expected to work in fostering forms of collaboration. Emerging patterns in the joint document were collaboratively refined with co‐authors through an iterative, dialogical approach, following the principles outlined by Francis Auton et al [35]. These discussions were documented and used by MF and HS to further develop the IPT regarding recurring patterns, contextual factors, and mechanisms explaining how and why the intervention worked in each case. In a final discussion among all authors, the group agreed that five preliminary ICAMO configurations formed the initial programme theory. This IPT outlined how the intervention—a facilitated, structured co‐design process—was assumed to trigger mechanisms such as trust, mutual understanding, ownership, and engagement, ultimately leading to the long‐term outcome. This outcome was articulated as strengthened organisational relationships and sustainable forms of collaboration within the context of motivated yet organisationally separate actors.

#### 2.4.2 Stage 2: Testing and Refining the Initial Programme Theory

To test and refine the IPT, six group interviews were conducted by authors MF and HS. For Case A, the interviews were conducted 16 months after the workshop series, for Case B 12 months, and for Case C 14 months. In Cases A and C, group interviews were held separately with third sector and social services representatives (Case A *n* = 2, Case C *n* = 3). In Case B, one joint interview was conducted due to scheduling challenges and staff turnover. No primary care representatives participated in Stage 2 interviews. In total, nine representatives were interviewed in Case A, five in Case B, and 11 in Case C. Sessions lasted 70–90 min. A realist interview technique was applied, based on the teacher‐learner cycle [22, 36]. The core team first presented the IPT (acting as ‘teachers’) and then (acting as ‘learners’), invited participants to reflect, question, and co‐construct explanations. This approach encouraged critique and refinement of the IPT and its ICAMO configurations, making participants active contributors to sense‐making and theory development. Initially, the participants in Cases A and B were asked to consider five preliminary ICAMO configurations. As a result of the analysis of Case A and B interviews and the following analytical discussions among the authors, these five configurations were reduced to three through further abstraction. In the analysis, the outcomes were revised, from the initial long‐term outcome of “strengthened organisational relationships and sustainable forms of collaboration” to immediate outcomes emerging from the co‐design process. Participants in Case C were subsequently asked to reflect on the three ICAMOS.

Throughout the analysis, authors MF and HS used structured memos (inspired by Gilmore et al [37]) to capture emerging insights and decisions on refining ICAMOs. All group interviews were transcribed and analysed deductively [38] by authors MF and HS using the IPT to fine‐tune and validate the programme theory. In the analysis, statements that were in alignment with the ICAMO configurations were provided with a code that indicated the level of alignment. Statements that did not align with the ICAMO configurations were coded inductively. All codes, both the deductively, i.e., in alignment with the ICAMOs, and the inductively generated, were compiled into an excel file that provided an overview over the five ICAMOs (for the analysis of Cases A and B) and later the three ICAMOs (for the analysis of Case C). The overview was discussed between the authors MF and HS and in the research group. This process resulted in a refined programme theory, which includes three ICAMOs.

## 3 Results

**Figure 1:**
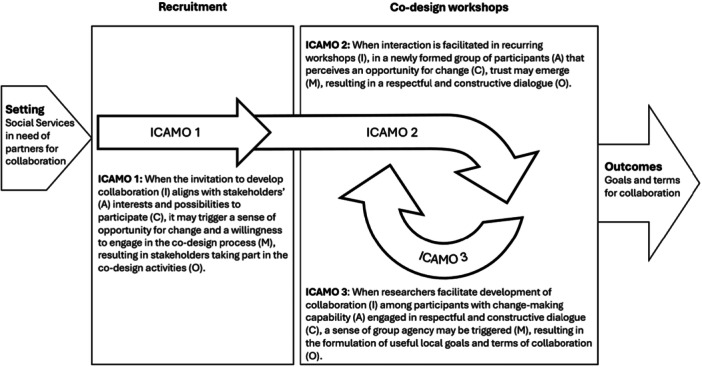
Illustration of the conceptual linkage of the three interconnected ICAMO configurations.

The analysis identified three interconnected ICAMO configurations (Figure 1) reflecting contextual factors and mechanisms within a co‐design process aimed at developing collaboration between social services, primary care and the third sector. ICAMO1 illustrates factors influencing stakeholders' decision to join the co‐design process, leading to ICAMO2 which captures how stakeholders begin forming a group and engaging in constructive dialogue. Once this dialogue is established, it shapes the context for the next ICAMO (ICAMO3), which shows how it deepens the stakeholders' sense of being a collaborative group capable of creating conditions for joint work. This experience reinforces trust within the group (ICAMO2), further strengthening collaboration and its prerequisites.

In the following sections, we elaborate on each ICAMO configuration, highlighting the contextual factors that mattered and how, together with different aspects of facilitation, they shaped the mechanisms that were triggered (or failed to be triggered), leading to desired or undesired outcomes that shaped the prerequisites for collaboration.

### 3.1 ICAMO 1

When the invitation to develop collaboration (I) aligns with stakeholders' (A) interests and possibilities to participate (C), it may trigger a sense of opportunity for change and a willingness to engage in the co‐design process (M), resulting in stakeholders taking part in the co‐design activities (O).

#### 3.1.1 Intervention and Actors

Initially, researchers and municipal Prevention units in each case identified relevant local stakeholders who could potentially collaborate to prevent deterioration of health among community‐dwelling older adults and, therefore, should be invited to the co‐design process. The invitations were formulated in collaboration with contact persons at the Prevention units to ensure contextual fit. They were then emailed to stakeholders, either to a manager or to an individual known to the contact person, and included details on workshop dates, times, and locations, the purpose of the workshops, the invited organisations, and brief information about research participation. Stakeholders then decided internally who would represent their organisation.

#### 3.1.2 Context

All stakeholders invited to the co‐design process expressed interest in working proactively to promote older adults' health and to collaborate in this effort, though their motivations and levels of commitment varied. Social services emphasised that collaboration with primary care and the third sector aligned with their formal organisational mission. They also linked their participation to making better use of tax‐funded resources for instance by collaborating to develop new activities at a senior citizen centre (Case A), by developing work routines for a newly established function within the Prevention unit to guide older adults in navigating available support interventions (Case B), or develop local volunteer activities for older adults and link them more closely to the municipality's preventive activities (Case C). The third sector organisations were keen to meet their members' interests regarding both prevention and health promotion, while positioning themselves as respected collaborative partners to social services and primary care. Senior citizens' associations also found it important to join when peer organisations were involved, to ensure equal access to opportunities and potential benefits for their members. Across all organisations, the individual participants viewed the co‐design process as an opportunity to develop their networks.

I really wanted to join, but I wasn't entirely sure what it would be about. […] Still, of course I wanted to be involved when it concerns my local community. And then there's the networking aspect, of course, because I don't yet have the same network as many of my peers [in my organisation].

(Third sector representative, Case A)

Previous experiences of collaboration, both positive and negative, shaped stakeholders' interest in new partnerships and influenced their response to the invitation. In Cases A and C, previous successful projects or an invitation from a well‐known, trusted, and capable person (such as the head of the Prevention unit) increased third sector stakeholders' willingness to participate. Third sector stakeholders with negative experiences referred to past situations of unequal power balance between organisations and repeatedly being neglected in attempts to collaborate. However, given that the invitation came from social services, considered an important actor in preventive work, and that the process included an external facilitator who could help balance power differentials, most stakeholders saw the arrangement as an opportunity for a different, more constructive process. Others emphasised that what sparked their interest was the research context and the fact that the co‐design was led by researchers, which they considered a guarantee of a systematic and evidence‐based approach. The invited organisations also considered whether they had the practical possibilities, i.e., logistics and resources, to participate in 12 h of workshops (on the given dates and times) during the forthcoming months. Several interested organisations declined due to short notice, scheduling conflicts, or limited staffing. In all cases, it was evident that primary care's main challenge in prioritising participation stemmed from the lack of financial flexibility in the reimbursement system, which restricted their ability to allocate staff for collaborative activities. In most third sector organisations, where activities largely rely on voluntary engagement, the barrier was instead tied to individuals' personal priorities.

#### 3.1.3 Mechanism and Outcomes

Alignment of the invitation with favourable contextual conditions triggered *a sense of opportunity for change and a willingness to engage in the co‐design process* among the stakeholders. This included both intentions and expectations of new and improved conditions for collaboration, and a notion that the timing was right.

Me and my manager ‐ we felt we just couldn't get things moving [at the senior citizen centre] despite all the work we had put in. But [the researchers' proposal] pushed us, and we thought, ‘this would be great’, and we started thinking about who we could involve.

(Social services representative, Case A)

In most participating organisations, a sense of opportunity for change was evident at multiple levels, both among individual participants and at the management or steering‐group level. However, in one of the stakeholder organisations in Case A, a sense of opportunity and willingness to engage was triggered at the organisational level, whereas the appointed representative who participated was not fully convinced of the intervention's potential. In Case C, the opposite occurred, an individual participant personally perceived an opportunity for change and joined on their own initiative, without clearly anchoring it within their home organisation.

### 3.2 ICAMO2

**When interaction is facilitated in recurring workshops (I), in a newly formed group of participants (A) that perceives an opportunity for change (C), trust may emerge (M), resulting in a respectful and constructive dialogue (O).**

#### 3.2.1 Intervention and Actors

During the workshops, the facilitators aimed to create opportunities for interpersonal interaction that would help participants get to know each other and develop a better understanding of one another's organisations. To foster a climate in which participants felt encouraged to express their views, listen actively, and reflect on their assumptions and biases, several facilitation methods were used. These included playful elements such as icebreaker activities at the beginning of each workshop and informal exercises during the coffee breaks. The facilitation also involved documenting and moderating group sessions to ensure that all participants had the opportunity to speak, as well as confirming, feeding back and reflecting on participants' statements and group discussions, and summarising the content of small‐group discussions for the whole group. Participants were frequently divided into cross‐organisational pairs or groups to build relationships and to learn about each other's conditions and expectations regarding collaboration. Since the workshops were held regularly over a 4–6‐month period, the facilitation was adapted over time to support the gradual development of relationships within the group.

#### 3.2.2 Context

Participants in all three cases expressed curiosity and anticipation that the process would lead to change, at least in terms of personal knowledge and strengthened relationships. However, some were sceptical about whether the co‐design process would bring about real and sustainable change in community prevention work, referencing past experiences where collaboration initiatives had fizzled out.

The newly formed co‐design groups consisted of individuals with varying levels of familiarity with each other and each other's organisations. The settings in Cases A and C were smaller and more cohesive communities with a limited number of third sector and primary care organisations operating locally. Hence, in those cases, most participants already knew each other to some extent, and their organisations had collaborated previously. These groups remained highly stable throughout the process, with most participants consistently attending each session. In contrast, Case B involved a significantly larger community, with a diverse mix of private and public primary care and social care providers, as well as numerous third sector organisations serving older adults. Fewer participants in this case were familiar with each other compared to the other cases, and continuity in participation was also weaker in this group.

You know, [this municipality] is big—there are so many primary health care centres and different home care services everywhere.

(Third sector representative, Case B)

#### 3.2.3 Mechanism and Outcome

The workshops were led by researchers (authors MF, HS, KJ) acting as external, impartial facilitators. Participants perceived this as allowing them to step outside their formal organisational roles and engage in co‐design on an equal footing, without concerns about hidden agendas, while still being able to contribute their respective expertise. The facilitators' external and impartial status, without strong ties or loyalties to local stakeholders, was considered particularly valuable by Prevention unit managers, who typically organise and lead collaborative initiatives and, therefore, hold greater formal influence. This influence can be difficult to balance when the aim is to foster a dialogic climate in which stakeholders from different sectors and with varying conditions can contribute and thrive.

We wanted to be part of it on the same terms as everyone else, so it wouldn't automatically be the municipality running everything. It was important that you [i.e. the researchers] were the ones facilitating the process. It felt good to be on an equal footing, because then you can interact in a different way.

(Social services representative, Case A)

Temporarily dismantling hierarchical structures and entrenched roles within the co‐design sessions created opportunities for more informal conversations. As the groups met regularly over an extended period, they began to develop relationships that encompassed both professional and personal dimensions. As participants got to know each other and built stronger connections, a sense of trust emerged within the group. They explained that the facilitation enabled them to engage in discussions without “having to fight to be heard”, allowing them to focus on listening and responding to others' perspectives. Participants also described how affirmation received from both facilitators and fellow participants made them feel acknowledged and valued.

The mechanism of trust was activated to varying degrees across the cases, leading to different outcomes in terms of respectful and constructive dialogue. When fewer participants were familiar with each other beforehand, the process of building trust appeared to be more difficult. In Case B, which involved a more diverse mix of stakeholders than the other cases, considerable time was spent reporting and discussing organisational differences and stakeholder‐specific challenges. Here, the recurring workshops over an extended period appear to have been particularly important for fostering trust.

I think it really helped that we already knew, like, ‘Okay, we're meeting again on this date.’ Just having something scheduled and knowing what we'd talk about made a big difference. It gave a sense that this would keep going, and that probably helped build relationships.

(Social services representative, Case B)

Cases A and C included participants representing stakeholders with more established connections and prior experiences of collaboration, both positive and negative. In these cases, building trust and fostering a respectful, constructive climate for dialogue proved smoother.

It felt very respectful because someone was guiding the process, and I think we all committed from the start to be mindful of how we spoke to each other so it wouldn't turn into an ‘us and them’ situation. We really had to work on that—I noticed it throughout the process. It's something that has actually changed over time. Today, people have a completely different attitude and choose their words much more carefully.

(Social services representative, Case C)

### 3.3 ICAMO3

**When researchers facilitate development of collaboration (I) among participants with change‐making capability (A) engaged in respectful and constructive dialogue (C), a sense of group agency may be triggered (M), resulting in the formulation of useful local goals and terms of collaboration (O).**

#### 3.3.1 Intervention and Actors

Facilitating the ideation and prototyping of collaboration aimed to build a shared understanding of the problem and to define common goals and terms for collaboration, such as meeting frequency, agendas, and responsibilities. The workshops incorporated a variety of creative activities in pairs, small groups, or the whole group, including brainstorming, brainwriting, worst‐possible‐idea exercises, and open voting to decide which ideas to pursue. Facilitators typically supported the process by moderating and documenting pair or small‐group discussions. In some cases, participants volunteered or were assigned to take on this role. Group work outcomes were summarised and reported back to the larger group by either facilitators or participants. The facilitation aimed to support participants' sense‐making throughout the co‐design process by continuously describing and summarising the process and its outcomes. Between workshops, the facilitation team compiled and analysed the overall results and shared them with participants via email. Results were also sent to managers in participating organisations, as they were considered crucial for integrating the outcomes into local operations. Each workshop began with facilitators presenting a summary of the previous session, providing opportunities for participants to discuss, validate, and share any feedback received from their home organisations.

#### 3.3.2 Context

A central contextual aspect influencing the activation of the third mechanism, operating in a ripple effect manner, was the dialogue climate, which varied across cases in terms of mutual respect and focus on constructive interaction (described in detail in ICAMO2). Beyond dialogue, participants' capability to initiate, support or influence change was another key factor, operating in two main ways. First, it involved participants acting as organisational representatives during the workshops and contributing perspectives based on their expertise. This expertise encompassed organisational knowledge, operational insight, system awareness, and personal competencies shaped by diverse educational, professional and experiential backgrounds. Second, the capability also included sharing information and anchoring co‐design results within their home organisations. Participants with a formal mandate to influence development in their home organisations—such as managers or chairs—were generally better positioned to drive change than those in roles with less organisational authority. For example, board members of senior citizens' associations and managers of social service organisations in Cases A and C introduced a permanent agenda item dedicated to sharing information from the co‐design process. In contrast, participants without a formal mandate or opportunities to influence decision‐making in their home organisations, such as staff representatives from home care and primary care in Case B, had to rely on indirect strategies, including posting written updates about the process and its results in staff rooms. Because their capability to initiate change was limited, the facilitation team convened a meeting with managers from the participating organisations to brief them on the process and support anchoring of results at an appropriate decision‐making level. However, even in organisations where permanent agenda items were successfully introduced, participants often struggled to convey what the co‐design process entailed to their colleagues who were unfamiliar with co‐design and sometimes with details of the other participating organisations. Some participants developed strategies to adapt and package the information to make it more accessible and relevant for their colleagues.

What I've done is gather information from two workshops and then put together something bigger—like, here's what has happened so far and what's coming next. That way it's easier for my colleagues to grasp.

(Municipal representative, Case C)

#### 3.3.3 Mechanism and Outcome

One intended outcome of the co‐design process was the formulation of useful local goals and terms for collaboration. This outcome was enabled by a mechanism of group agency, which was activated when participants, given their capability to initiate and influence change, engaged in respectful and constructive dialogue. Through this process, they developed an emergent belief that they, as a group, had the legitimacy and collective capability to act. This mechanism, in turn, made it possible for them to formulate meaningful and jointly owned goals and terms for collaboration.

In the end, it became a ‘we’—not a ‘what are they doing?’ or ‘what are you going to do to fix this?’

(Social services representative, Case C)

The mechanism was clearly triggered in Cases A and C, but only weakly in Case B. In Case B, the ICAMO2 pattern produced a more ambiguous result: the dialogue was less constructive, participants formed weaker connections than in the other two cases, and, in addition, a larger share of participants had limited capability to initiate or influence change. Primarily, this was understood as resulting from Case B being a more diverse group than Cases A and C. In Case B, the participants were less familiar with each other, had less participant continuity in the co‐design, and the community had a more diverse mix of primary care and social care providers. Moreover, in Case B, no manager participated in the co‐design process, which may have limited the opportunities for the group to develop a sense of group agency. In addition, as few participants held a formal mandate this reduced their ability to transfer shared understanding from the co‐design group to their home organisations. This combination of factors was interpreted as explaining why the mechanism of group agency was not activated to the same extent.

Across all three cases, participants formulated common local goals and agreed on terms for collaboration. However, the perceived benefits of these arrangements varied. In Cases A and C, the groups continued to work according to the agreed goals and terms after the co‐design process ended. In Case B, the group retained similar meeting formats but almost immediately abandoned the agreed‐upon goal and agenda developed during the co‐design process. This indicates that the co‐design output was not perceived as equally useful or applicable in the local context compared with the other two cases. One possible explanation is that the underlying mechanism was less strongly activated in Case B, reducing the extent to which participants felt ownership of, or commitment to, the co‐designed goal.

## 4 Discussion

In this study, we conducted a realist process evaluation of a co‐design process in three cases. In these cases, representatives from municipal and social services, primary care, and third sector organisations jointly formulated shared goals and terms of collaboration to develop local work on prevention and health promotion targeting older adults living at home. The aim of the study was to evaluate contextual factors and mechanisms of importance in such a co‐design process. Three interconnected ICAMO configurations were identified. The first concerns stakeholders' assessment of the potential value of participating in the co‐design process and their decision to engage. The second highlights how emerging trust enabled respectful and constructive dialogue. The third focuses on the facilitation of ideation and prototyping of goals and terms for collaboration.

As shown in ICAMO1, a key aspect of creating favourable conditions for co‐design is identifying and involving the “right” people. Ideally, individuals should represent relevant perspectives on the focus area and be both motivated and able to contribute constructively [10]. Moreover, a key feature of co‐design is the involvement of service users with their own, lived experience of the focus area [10]. Despite this recognised importance [39], recruitment strategies are rarely reported in design studies [40]. In this project, researchers asked municipal Prevention units to invite actors they wished to engage. Each invited organisation then independently selected individuals to represent them in the co‐design process. This multi‐step recruitment strategy resulted in considerable variation in participants' familiarity with each other, as well as roles and mandate to drive change within their organisations. Such a recruitment approach entails both risks and advantages. On the one hand, people tend to prefer what is familiar [41], which may lead to inviting only well‐known organisations or individuals, limiting diversity of perspectives. On the other hand, existing familiarity among participants in the co‐design group may provide a head start, as prior relationships often support collaboration [7]. In our study, the case with the fewest prior connections and more participants without mandate to drive change (Case B) appeared to experience less favourable outcomes compared to the two other cases. When organisations select their own representatives, clear communication about what is expected from them is crucial to ensure participants are prepared and equipped to contribute [42]. Our findings, therefore, suggest that recruiting participants who are already familiar within the system and who hold a formal mandate or agency for change may better support the establishment of collaborative processes. At the same time, previous research underscores the importance of not shying away from the challenge of involving new stakeholders, as they can contribute perspectives and expertise that support more innovative and relevant solutions [10]. Furthermore, invitations that clearly articulate the significance of participants' characteristics and convey a sense of opportunity for practical change may help increase engagement and attract a broader range of actors who are willing to contribute to change efforts. Previous research has shown that trust between collaborating parties is a crucial factor for effective and sustainable collaboration [5, 7, 26]. This is hardly surprising: trust encourages and is reinforced by information sharing and open communication, and it reduces perceived risks associated with dependence on others, as well as fears that other actors might exploit collaborative efforts [26, 43]. Moreover, trust and communication are central to reducing conflicts and misunderstandings in collaboration. Trustful personal relationships give partners the confidence to raise problems and criticism and strengthen the motivation to resolve conflicts constructively [44]. This study adds evidence that trust is essential for developing collaboration. As described in ICAMO2, trust began to emerge among participants in all three cases when facilitation enabled discussions that allowed space to consider and respond to each other's perspectives. As Knowles et al. [45] demonstrated, our analysis shows that beyond facilitation, an open and trustful atmosphere contributed to participants' ability to explore tensions constructively and reconsider preconceived notions about one another. Trust has been identified as a key mechanism for achieving co‐design outcomes, as highlighted in a realist review [27] and a recent realist synthesis [46]. In the latter, it is described as ‘alignment’, involving the process of sharing voices, feeling heard, and the bringing together of diverse perspectives and ideas [46]. Trust thus appears to be crucial for both developing and maintaining collaboration by creating an open environment that fosters innovation and leads to more sustainable solutions. The cyclical and self‐reinforcing role of trust in nurturing collaboration, called the ‘trust building loop’ [26, 43], therefore, underscores the importance of allocating time and resources during co‐design to foster trust [7].

Our findings highlight the role of external facilitators in enabling trust and constructive dialogue, which in turn created a context where stakeholders could develop collaboration. Recent literature reviews emphasise several key targets for facilitators, similar to those in our study, such as building trusting relationships, promoting power sharing, and facilitating communication [14, 39, 46]. Furthermore, Masterson et al. [46] highlights that iterative interaction with mechanisms of documentation, i.e., transparent recording and sharing of information, and dialogue, may lead to the development of trust that in turn enables outcomes in terms of agreement on goals and ways of working. This aligns with our study, where facilitators analysed and summarised results between workshops, shared documentation with participants, and used these summaries to guide discussions in subsequent workshops, as described in ICAMO3, contributing to sensemaking and fostering a shared sense of agency within the group. Facilitators' ability to support power sharing, communication, and trust are important aspects of facilitating co‐design [14] and this multi‐faced role often requires multiple facilitators [42]. This study contributes to the literature by suggesting that not only the facilitators' intended goals and activities matter, but also their perceived impartiality. This aspect has been highlighted only sparingly in previous research [14]. In our study, the perceived neutrality of the external facilitators among participants helped alleviate concerns about hidden agendas influencing the process or its outcomes. Such impartiality may be particularly important when participants are developing terms of collaboration that they themselves will use and sustain after the co‐design process ends, rather than producing an output intended for broader use in other contexts by others. Supporting the development of collaboration should, therefore, involve building trusting relationships not only among participants [14, 39, 46] but also between facilitators and participants.

Our findings point to several important implications for future co‐design initiatives. First, facilitators need to be perceived as impartial in relation to involved stakeholders, as such neutrality appears central for establishing a climate of trust. Second, constructive participation seems to require representatives who combine practical knowledge with a genuine motivation for change and who have the mandate or organisational conditions to act on the outcomes. Finally, the results underscore the importance of continuity over time: a sufficiently stable group is critical both for maintaining the necessary competence and for building relationships and sustaining trust throughout the co‐design process.

### 4.1 Methodological Considerations

A limitation of this study is the absence of perspectives from certain stakeholders. First, we were unable to conduct interviews with actors who declined the invitation to participate in the co‐design process, which may have influenced the findings related to ICAMO1 and our understanding of what affects stakeholders' willingness to engage. Second, interviews to test and refine the IPT were conducted only with a subset of co‐design participants. Although we intended to include primary care representatives, they were unable to participate due to time constraints and limited availability. This may have influenced findings for ICAMO configurations 2 and 3. Another consideration is that some authors of this realist process evaluation were also involved in conducting the co‐design process. On the one hand, this dual role allowed us to ask more informed and contextually relevant questions, drawing on in‐depth knowledge of the process. On the other, existing guidelines recommend that process evaluation teams remain separate from intervention teams [17] to reduce the risk of social desirability bias in participants' responses during interviews. However, the most significant methodological challenge in our study was distinguishing between mechanisms and contextual factors during analysis, an issue commonly reported in realist evaluations [47]. Although the distinction between these concepts has been described in theory [48, 49], in practice the boundaries between context and mechanism are often blurred, making it difficult to clearly define how these concepts relate to each other and to outcomes. Our experience suggests that while theoretical definitions provide guidance, applying them in complex, real‐world settings require iterative interpretations and transparency about analytical decisions.

### 4.2 Conclusion

This study identified key contextual factors and mechanisms in a co‐design process aimed at fostering collaboration among stakeholders in social services, primary care, and the third sector. Successful co‐design processes require facilitation that is both sensitive to contextual conditions and perceived as impartial, as such neutrality is essential for establishing trust. Constructive participation further depends on representatives who have relevant practical knowledge, a genuine sense of opportunity for change. Moreover, representatives must have the mandate or organisational conditions to act on outcomes in their home organisations, thereby supporting group agency. Finally, ensuring continuity in participation by keeping the same individuals engaged throughout the process is crucial for maintaining competence within the group. Continuity is key to strengthening the relationships and trust needed to build shared goals and collaborative structures.

## Author Contributions

**Maria Flink:** conceptualisation (lead), investigation (lead), formal analysis (lead), methodology (lead), visualisation (lead), writing – original draft (lead), writing – review and editing (lead). **Karin Johansson:** investigation (equal), formal analysis (support), writing – review and editing (equal). **Christofer Lindgren, Sarah Wallcook, Charlotte Klinga** and **Bettina Meinow:** investigation (support), formal analysis (support), writing – review and editing (equal). **Ida Goliath:** funding acquisition (lead), conceptualisation (equal), investigation (equal), formal analysis (support), writing – review and editing (lead), supervision (lead). **Helena Strehlenert:** conceptualisation (lead), investigation (lead), formal analysis (lead), methodology (lead), visualisation (lead), writing – original draft (lead), writing – review and editing (lead).

## Ethics Statement

Ethical approval was granted by the Swedish Ethical Review Authority (registration number 2021‐04645).

## Consent

All participants were informed about the study before providing informed consent.

## Conflicts of Interest

The authors declare no conflicts of interest.

## Data Availability

The data that support the findings of this study are available from the corresponding author upon reasonable request.
